# 5-Hy­droxy-7-meth­oxy-2-methyl-4*H*-chromen-4-one from *Dysoxylum macrocarpum* (Meliaceae)

**DOI:** 10.1107/S1600536810025146

**Published:** 2010-07-03

**Authors:** Ibrahim A. Najmuldeen, Abdul Hamid Abdul Hadi, Khalijah Awang, Khalit Mohamad, Seik Weng Ng

**Affiliations:** a Department of Chemistry, University of Malaya, 50603 Kuala Lumpur, Malaysia; b Department of Pharmacy, Faculty of Medicine, University of Malaya, 50603 Kuala Lumpur, Malaysia

## Abstract

Both independent mol­ecules in the asymmetric unit of the title compound, C_11_H_10_O_4_, are almost planar (r.m.s. deviations = 0.011 and 0.033 Å). In both mol­ecules, the hy­droxy group is intra­molecularly hydrogen bonded to the ketonic O atom. The independent mol­ecules are stacked alternately along the *a* axis, with the centroids of their chromene ring separated by distances of 4.490 (1) and 3.621 (1) Å.

## Related literature

For studies on other *Dysoxylum* species, see: Ismail *et al.* (2009 ▶); Lakshmi *et al.* (2007 ▶); Mohamad *et al.* (1999 ▶); Mohanakumara *et al.* (2010 ▶); Senthil Nathan *et al.* (2008 ▶); Xie *et al.* (2008 ▶).
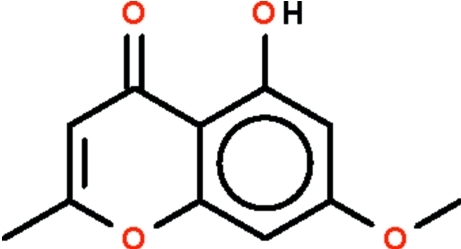


## Experimental

### 

#### Crystal data

C_11_H_10_O_4_
*M* *_r_* = 206.19
Monoclinic, 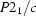

*a* = 7.7393 (3) Å
*b* = 14.5373 (6) Å
*c* = 16.8263 (7) Å
β = 98.848 (1)°
*V* = 1870.57 (13) Å^3^
*Z* = 8
Mo *K*α radiation
μ = 0.11 mm^−1^
*T* = 100 K
0.35 × 0.35 × 0.02 mm

#### Data collection

Bruker SMART APEXII area-detector diffractometer
17796 measured reflections
4285 independent reflections
3795 reflections with *I* > 2σ(*I*)
*R*_int_ = 0.017

#### Refinement

*R*[*F* ^2^ > 2σ(*F* ^2^)] = 0.035
*wR*(*F* ^2^) = 0.108
*S* = 1.02
4285 reflections
283 parameters
2 restraints
H atoms treated by a mixture of independent and constrained refinement
Δρ_max_ = 0.34 e Å^−3^
Δρ_min_ = −0.28 e Å^−3^

### 

Data collection: *APEX2* (Bruker, 2009 ▶); cell refinement: *SAINT* (Bruker, 2009 ▶); data reduction: *SAINT*; program(s) used to solve structure: *SHELXS97* (Sheldrick, 2008 ▶); program(s) used to refine structure: *SHELXL97* (Sheldrick, 2008 ▶); molecular graphics: *X-SEED* (Barbour, 2001 ▶); software used to prepare material for publication: *publCIF* (Westrip, 2010 ▶).

## Supplementary Material

Crystal structure: contains datablocks global, I. DOI: 10.1107/S1600536810025146/ci5120sup1.cif

Structure factors: contains datablocks I. DOI: 10.1107/S1600536810025146/ci5120Isup2.hkl

Additional supplementary materials:  crystallographic information; 3D view; checkCIF report

## Figures and Tables

**Table 1:** Hydrogen-bond geometry (Å, °)

*D*—H⋯*A*	*D*—H	H⋯*A*	*D*⋯*A*	*D*—H⋯*A*
O3—H3⋯O2	0.85 (1)	1.83 (1)	2.618 (1)	154 (2)
O7—H7⋯O6	0.85 (1)	1.79 (1)	2.595 (1)	156 (2)

**Fig. 1.**
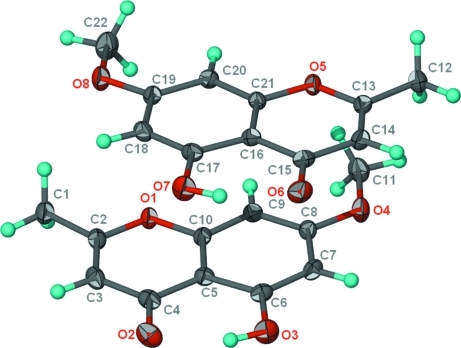
Displacement ellipsoid plot (Barbour, 2001) of the two independent molecules of C11H10O4 at the 70% probability level. H atoms are drawn as spheres of arbitrary radii.

## supplementary crystallographic information

### Comment

*Dysoxylum *produces terpenes, terpenoids and other compounds, as noted in studies on *Dysoxylum acutangulum*, *Dysoxylum beddomei*, *Dysoxylum binectariferum*, *Dysoxylum densiflorum*, *Dysoxylum malabaricum* and *Dysoxylum macranthum* (Ismail *et al.*, 2009; Lakshmi *et al.*, 2007; Mohamad *et al.*, 1999; Mohanakumara *et al.*, 2010; Senthil Nathan *et al.*, 2008; Xie *et al.*, 2008). A coumarin (Scheme I) is isolated from *Dysoxylum macrocarpum* in the present study. There are two independent molecules (Fig. 1). Both independent molecules are planar [r.m.s. deviations 0.011 and 0.033 Å); one is stacked over the other [dihedral angle between the planes 4.5 (1) °] but the distance between them exceeds 3.5 Å. The hydroxy group is intramolecularly hydrogen bonded to the ketonic oxygen atom.

### Experimental

Dried ground leaves of *Dysoxylum macrocarpum *(1.4 kg) were soaked in hexane for three days. The solvent was removed and the plant material was dried; the dried plant material was extracted with dichloromethane for another three days. The dichloromethane was removed by evaporation to give a crude material (30 g) that was subjected to column chromatography over silica gel. Separation was effected with hexane-dichloromethane (1:0 to 0:1v/v); the polarity was increased with methanol. One fraction was eluted with hexane-methanol (92:8 *v*/*v*) to give a compound that was further purified by silica gel column chromatography with hexane-ethyl acetate (70:30 *v*/*v*) to yield colourless crystals.

### Refinement

Carbon-bound H-atoms were placed in calculated positions (C–H = 0.95–0.98 Å) and were included in the refinement in the riding model approximation, with *U_iso_*(H) set to 1.2 to 1.5*U_eq_*(C). The hydroxy H-atoms were located in a difference Fourier map, and were refined with a distance restraint of O–H = 0.84 (1) Å; their U_iso_ parameters were freely refined.

### Crystal data

**Table:**

C_11_H_10_O_4_	*F*(000) = 864
*M**_r_* = 206.19	*D*_x_ = 1.464 Mg m^−^^3^
Monoclinic, *P*2_1_/*c*	Mo *K*α radiation, λ = 0.71073 Å
Hall symbol: -P 2ybc	Cell parameters from 9622 reflections
*a* = 7.7393 (3) Å	θ = 2.5–28.3°
*b* = 14.5373 (6) Å	µ = 0.11 mm^−^^1^
*c* = 16.8263 (7) Å	*T* = 100 K
β = 98.848 (1)°	Plate, colourless
*V* = 1870.57 (13) Å^3^	0.35 × 0.35 × 0.02 mm
*Z* = 8	

### Data collection

**Table:**

Bruker SMART APEXII area-detector diffractometer	3795 reflections with *I* > 2σ(*I*)
Radiation source: fine-focus sealed tube	*R*_int_ = 0.017
graphite	θ_max_ = 27.5°, θ_min_ = 1.9°
ω scans	*h* = −10→10
17796 measured reflections	*k* = −18→18
4285 independent reflections	*l* = −20→21

### Refinement

**Table:**

Refinement on *F*^2^	Primary atom site location: structure-invariant direct methods
Least-squares matrix: full	Secondary atom site location: difference Fourier map
*R*[*F*^2^ > 2σ(*F*^2^)] = 0.035	Hydrogen site location: inferred from neighbouring sites
*wR*(*F*^2^) = 0.108	H atoms treated by a mixture of independent and constrained refinement
*S* = 1.02	*w* = 1/[σ^2^(*F*_o_^2^) + (0.0679*P*)^2^ + 0.4625*P*] where *P* = (*F*_o_^2^ + 2*F*_c_^2^)/3
4285 reflections	(Δ/σ)_max_ = 0.001
283 parameters	Δρ_max_ = 0.34 e Å^−^^3^
2 restraints	Δρ_min_ = −0.28 e Å^−^^3^

### Fractional atomic coordinates and isotropic or equivalent isotropic displacement parameters (Å^2^)

**Table:**

	*x*	*y*	*z*	*U*_iso_*/*U*_eq_	
O1	0.49963 (9)	0.31112 (5)	0.80755 (4)	0.01545 (16)	
O2	0.54836 (10)	0.03128 (5)	0.81510 (5)	0.02311 (18)	
O3	0.66721 (11)	0.03755 (5)	0.67769 (5)	0.02290 (18)	
O4	0.71726 (10)	0.32572 (5)	0.55887 (4)	0.01882 (17)	
O5	1.05826 (9)	0.38133 (5)	0.70570 (4)	0.01574 (16)	
O6	1.12544 (10)	0.10446 (5)	0.73680 (5)	0.02192 (17)	
O7	1.00123 (11)	0.11679 (5)	0.87114 (5)	0.02253 (18)	
O8	0.82751 (10)	0.40629 (5)	0.95136 (4)	0.01974 (17)	
C1	0.39489 (14)	0.32353 (7)	0.93075 (6)	0.0200 (2)	
H1A	0.3576	0.2864	0.9737	0.030*	
H1B	0.2964	0.3607	0.9049	0.030*	
H1C	0.4909	0.3641	0.9536	0.030*	
C2	0.45527 (12)	0.26180 (7)	0.87004 (6)	0.0163 (2)	
C3	0.46768 (13)	0.16950 (7)	0.87405 (6)	0.0184 (2)	
H3A	0.4329	0.1383	0.9186	0.022*	
C4	0.53257 (12)	0.11694 (7)	0.81228 (6)	0.0172 (2)	
C5	0.58040 (12)	0.17065 (6)	0.74648 (6)	0.0147 (2)	
C6	0.64722 (12)	0.12963 (7)	0.68075 (6)	0.0160 (2)	
C7	0.69215 (12)	0.18335 (7)	0.61950 (6)	0.0167 (2)	
H7A	0.7379	0.1559	0.5759	0.020*	
C8	0.66943 (12)	0.27902 (7)	0.62233 (6)	0.0148 (2)	
C9	0.60437 (12)	0.32217 (6)	0.68517 (6)	0.01419 (19)	
H9	0.5895	0.3870	0.6864	0.017*	
C10	0.56218 (12)	0.26615 (6)	0.74610 (5)	0.01350 (19)	
C11	0.69519 (14)	0.42341 (7)	0.55749 (6)	0.0205 (2)	
H11A	0.7287	0.4483	0.5079	0.031*	
H11B	0.7693	0.4506	0.6041	0.031*	
H11C	0.5725	0.4383	0.5596	0.031*	
C12	1.16596 (14)	0.38672 (7)	0.58240 (6)	0.0200 (2)	
H12A	1.2153	0.3477	0.5440	0.030*	
H12B	1.2512	0.4340	0.6033	0.030*	
H12C	1.0590	0.4162	0.5553	0.030*	
C13	1.12406 (12)	0.32926 (7)	0.65014 (6)	0.0163 (2)	
C14	1.14769 (13)	0.23789 (7)	0.65825 (6)	0.0180 (2)	
H14	1.1940	0.2047	0.6177	0.022*	
C15	1.10416 (12)	0.18917 (7)	0.72722 (6)	0.0164 (2)	
C16	1.03515 (12)	0.24579 (6)	0.78581 (6)	0.01414 (19)	
C17	0.98490 (12)	0.20810 (6)	0.85677 (6)	0.0159 (2)	
C18	0.91807 (12)	0.26380 (7)	0.91094 (6)	0.0167 (2)	
H18	0.8858	0.2386	0.9586	0.020*	
C19	0.89823 (12)	0.35822 (7)	0.89482 (6)	0.0151 (2)	
C20	0.94688 (12)	0.39841 (6)	0.82650 (6)	0.01472 (19)	
H20	0.9348	0.4626	0.8167	0.018*	
C21	1.01372 (12)	0.34029 (6)	0.77342 (5)	0.01377 (19)	
C22	0.78973 (16)	0.50156 (7)	0.93552 (7)	0.0250 (2)	
H22A	0.7344	0.5275	0.9791	0.038*	
H22B	0.7103	0.5077	0.8845	0.038*	
H22C	0.8986	0.5347	0.9321	0.038*	
H3	0.634 (2)	0.0179 (12)	0.7204 (8)	0.061 (6)*	
H7	1.043 (3)	0.0968 (13)	0.8304 (9)	0.070 (6)*	

### Atomic displacement parameters (Å^2^)

**Table:**

	*U*^11^	*U*^22^	*U*^33^	*U*^12^	*U*^13^	*U*^23^
O1	0.0181 (3)	0.0157 (3)	0.0134 (3)	0.0013 (3)	0.0054 (3)	0.0001 (2)
O2	0.0254 (4)	0.0153 (4)	0.0293 (4)	0.0011 (3)	0.0063 (3)	0.0063 (3)
O3	0.0307 (4)	0.0125 (3)	0.0266 (4)	0.0021 (3)	0.0078 (3)	−0.0015 (3)
O4	0.0253 (4)	0.0179 (4)	0.0146 (3)	0.0024 (3)	0.0074 (3)	0.0023 (3)
O5	0.0205 (3)	0.0151 (3)	0.0125 (3)	−0.0002 (3)	0.0055 (3)	0.0011 (2)
O6	0.0276 (4)	0.0138 (3)	0.0246 (4)	0.0010 (3)	0.0045 (3)	−0.0030 (3)
O7	0.0336 (4)	0.0123 (3)	0.0226 (4)	0.0006 (3)	0.0071 (3)	0.0036 (3)
O8	0.0259 (4)	0.0179 (4)	0.0174 (4)	0.0017 (3)	0.0094 (3)	−0.0009 (3)
C1	0.0191 (5)	0.0263 (5)	0.0152 (5)	−0.0007 (4)	0.0048 (4)	−0.0019 (4)
C2	0.0131 (4)	0.0229 (5)	0.0129 (4)	−0.0009 (4)	0.0020 (3)	0.0014 (4)
C3	0.0175 (5)	0.0222 (5)	0.0157 (5)	−0.0006 (4)	0.0033 (4)	0.0049 (4)
C4	0.0141 (4)	0.0171 (5)	0.0198 (5)	−0.0005 (3)	0.0007 (3)	0.0032 (4)
C5	0.0135 (4)	0.0146 (4)	0.0157 (5)	0.0000 (3)	0.0013 (3)	0.0006 (3)
C6	0.0154 (4)	0.0139 (4)	0.0180 (5)	0.0009 (3)	0.0003 (3)	−0.0022 (3)
C7	0.0176 (5)	0.0173 (5)	0.0151 (4)	0.0018 (3)	0.0024 (3)	−0.0034 (3)
C8	0.0144 (4)	0.0182 (5)	0.0117 (4)	−0.0005 (3)	0.0014 (3)	0.0012 (3)
C9	0.0152 (4)	0.0126 (4)	0.0146 (4)	0.0004 (3)	0.0016 (3)	0.0008 (3)
C10	0.0127 (4)	0.0152 (4)	0.0127 (4)	0.0008 (3)	0.0020 (3)	−0.0014 (3)
C11	0.0251 (5)	0.0175 (5)	0.0200 (5)	0.0011 (4)	0.0064 (4)	0.0051 (4)
C12	0.0216 (5)	0.0232 (5)	0.0162 (5)	−0.0034 (4)	0.0059 (4)	0.0006 (4)
C13	0.0143 (4)	0.0209 (5)	0.0136 (4)	−0.0031 (3)	0.0023 (3)	−0.0024 (3)
C14	0.0178 (4)	0.0206 (5)	0.0164 (5)	−0.0015 (4)	0.0050 (4)	−0.0049 (4)
C15	0.0144 (4)	0.0157 (4)	0.0186 (5)	−0.0011 (3)	0.0007 (3)	−0.0030 (4)
C16	0.0140 (4)	0.0137 (4)	0.0143 (4)	−0.0010 (3)	0.0010 (3)	−0.0002 (3)
C17	0.0165 (4)	0.0137 (4)	0.0170 (5)	−0.0016 (3)	0.0008 (3)	0.0021 (3)
C18	0.0175 (5)	0.0178 (5)	0.0151 (4)	−0.0023 (4)	0.0029 (3)	0.0032 (3)
C19	0.0142 (4)	0.0175 (5)	0.0139 (4)	−0.0007 (3)	0.0030 (3)	−0.0022 (3)
C20	0.0164 (4)	0.0126 (4)	0.0152 (4)	0.0000 (3)	0.0025 (3)	0.0004 (3)
C21	0.0135 (4)	0.0156 (4)	0.0120 (4)	−0.0020 (3)	0.0014 (3)	0.0018 (3)
C22	0.0343 (6)	0.0178 (5)	0.0250 (5)	0.0052 (4)	0.0110 (4)	−0.0019 (4)

### Geometric parameters (Å, °)

**Table:**

O1—C2	1.3596 (11)	C7—H7A	0.95
O1—C10	1.3729 (11)	C8—C9	1.3888 (13)
O2—C4	1.2514 (12)	C9—C10	1.3870 (13)
O3—C6	1.3494 (11)	C9—H9	0.95
O3—H3	0.850 (9)	C11—H11A	0.98
O4—C8	1.3637 (11)	C11—H11B	0.98
O4—C11	1.4302 (12)	C11—H11C	0.98
O5—C13	1.3611 (11)	C12—C13	1.4886 (13)
O5—C21	1.3761 (11)	C12—H12A	0.98
O6—C15	1.2496 (12)	C12—H12B	0.98
O7—C17	1.3517 (11)	C12—H12C	0.98
O7—H7	0.852 (9)	C13—C14	1.3450 (14)
O8—C19	1.3612 (11)	C14—C15	1.4430 (14)
O8—C22	1.4319 (12)	C14—H14	0.95
C1—C2	1.4880 (13)	C15—C16	1.4480 (13)
C1—H1A	0.98	C16—C21	1.3956 (13)
C1—H1B	0.98	C16—C17	1.4212 (13)
C1—H1C	0.98	C17—C18	1.3780 (14)
C2—C3	1.3463 (14)	C18—C19	1.4030 (13)
C3—C4	1.4412 (14)	C18—H18	0.95
C3—H3A	0.95	C19—C20	1.3920 (13)
C4—C5	1.4486 (13)	C20—C21	1.3862 (13)
C5—C10	1.3955 (13)	C20—H20	0.95
C5—C6	1.4220 (13)	C22—H22A	0.98
C6—C7	1.3795 (14)	C22—H22B	0.98
C7—C8	1.4035 (13)	C22—H22C	0.98
			
C2—O1—C10	119.45 (8)	O4—C11—H11C	109.5
C6—O3—H3	104.5 (13)	H11A—C11—H11C	109.5
C8—O4—C11	117.45 (7)	H11B—C11—H11C	109.5
C13—O5—C21	119.70 (8)	C13—C12—H12A	109.5
C17—O7—H7	103.2 (14)	C13—C12—H12B	109.5
C19—O8—C22	117.32 (8)	H12A—C12—H12B	109.5
C2—C1—H1A	109.5	C13—C12—H12C	109.5
C2—C1—H1B	109.5	H12A—C12—H12C	109.5
H1A—C1—H1B	109.5	H12B—C12—H12C	109.5
C2—C1—H1C	109.5	C14—C13—O5	122.61 (9)
H1A—C1—H1C	109.5	C14—C13—C12	126.13 (9)
H1B—C1—H1C	109.5	O5—C13—C12	111.25 (8)
C3—C2—O1	122.67 (9)	C13—C14—C15	121.42 (9)
C3—C2—C1	126.45 (9)	C13—C14—H14	119.3
O1—C2—C1	110.87 (8)	C15—C14—H14	119.3
C2—C3—C4	121.49 (9)	O6—C15—C14	123.03 (9)
C2—C3—H3A	119.3	O6—C15—C16	121.83 (9)
C4—C3—H3A	119.3	C14—C15—C16	115.14 (9)
O2—C4—C3	122.89 (9)	C21—C16—C17	117.60 (8)
O2—C4—C5	122.03 (9)	C21—C16—C15	120.37 (9)
C3—C4—C5	115.08 (9)	C17—C16—C15	122.02 (9)
C10—C5—C6	117.53 (8)	O7—C17—C18	119.54 (9)
C10—C5—C4	120.14 (9)	O7—C17—C16	119.91 (9)
C6—C5—C4	122.32 (9)	C18—C17—C16	120.55 (9)
O3—C6—C7	119.31 (9)	C17—C18—C19	119.21 (9)
O3—C6—C5	120.21 (9)	C17—C18—H18	120.4
C7—C6—C5	120.48 (9)	C19—C18—H18	120.4
C6—C7—C8	119.23 (9)	O8—C19—C20	123.40 (9)
C6—C7—H7A	120.4	O8—C19—C18	114.27 (8)
C8—C7—H7A	120.4	C20—C19—C18	122.33 (9)
O4—C8—C9	123.04 (8)	C21—C20—C19	116.84 (8)
O4—C8—C7	114.59 (8)	C21—C20—H20	121.6
C9—C8—C7	122.37 (9)	C19—C20—H20	121.6
C10—C9—C8	116.85 (8)	O5—C21—C20	115.79 (8)
C10—C9—H9	121.6	O5—C21—C16	120.75 (8)
C8—C9—H9	121.6	C20—C21—C16	123.46 (8)
O1—C10—C9	115.31 (8)	O8—C22—H22A	109.5
O1—C10—C5	121.16 (8)	O8—C22—H22B	109.5
C9—C10—C5	123.53 (8)	H22A—C22—H22B	109.5
O4—C11—H11A	109.5	O8—C22—H22C	109.5
O4—C11—H11B	109.5	H22A—C22—H22C	109.5
H11A—C11—H11B	109.5	H22B—C22—H22C	109.5
			
C10—O1—C2—C3	−0.95 (13)	C21—O5—C13—C14	0.18 (14)
C10—O1—C2—C1	178.64 (7)	C21—O5—C13—C12	−179.19 (7)
O1—C2—C3—C4	1.16 (15)	O5—C13—C14—C15	−0.03 (15)
C1—C2—C3—C4	−178.35 (9)	C12—C13—C14—C15	179.24 (9)
C2—C3—C4—O2	178.74 (10)	C13—C14—C15—O6	−179.58 (9)
C2—C3—C4—C5	−0.66 (14)	C13—C14—C15—C16	−0.25 (14)
O2—C4—C5—C10	−179.40 (9)	O6—C15—C16—C21	179.72 (9)
C3—C4—C5—C10	0.00 (13)	C14—C15—C16—C21	0.38 (13)
O2—C4—C5—C6	0.38 (15)	O6—C15—C16—C17	−1.11 (15)
C3—C4—C5—C6	179.78 (8)	C14—C15—C16—C17	179.55 (8)
C10—C5—C6—O3	−179.79 (8)	C21—C16—C17—O7	179.27 (8)
C4—C5—C6—O3	0.42 (14)	C15—C16—C17—O7	0.08 (14)
C10—C5—C6—C7	0.16 (14)	C21—C16—C17—C18	−0.18 (14)
C4—C5—C6—C7	−179.63 (8)	C15—C16—C17—C18	−179.37 (8)
O3—C6—C7—C8	179.29 (9)	O7—C17—C18—C19	−178.73 (9)
C5—C6—C7—C8	−0.66 (14)	C16—C17—C18—C19	0.72 (14)
C11—O4—C8—C9	−1.20 (13)	C22—O8—C19—C20	5.00 (14)
C11—O4—C8—C7	179.17 (8)	C22—O8—C19—C18	−174.86 (9)
C6—C7—C8—O4	−179.82 (8)	C17—C18—C19—O8	178.59 (8)
C6—C7—C8—C9	0.55 (14)	C17—C18—C19—C20	−1.27 (14)
O4—C8—C9—C10	−179.53 (8)	O8—C19—C20—C21	−178.65 (8)
C7—C8—C9—C10	0.08 (14)	C18—C19—C20—C21	1.19 (14)
C2—O1—C10—C9	−179.84 (8)	C13—O5—C21—C20	−179.66 (8)
C2—O1—C10—C5	0.26 (13)	C13—O5—C21—C16	−0.03 (13)
C8—C9—C10—O1	179.48 (7)	C19—C20—C21—O5	178.99 (8)
C8—C9—C10—C5	−0.61 (14)	C19—C20—C21—C16	−0.62 (14)
C6—C5—C10—O1	−179.60 (8)	C17—C16—C21—O5	−179.46 (8)
C4—C5—C10—O1	0.19 (14)	C15—C16—C21—O5	−0.26 (14)
C6—C5—C10—C9	0.50 (14)	C17—C16—C21—C20	0.13 (14)
C4—C5—C10—C9	−179.71 (9)	C15—C16—C21—C20	179.34 (9)

### Hydrogen-bond geometry (Å, °)

**Table:**

*D*—H···*A*	*D*—H	H···*A*	*D*···*A*	*D*—H···*A*
O3—H3···O2	0.85 (1)	1.83 (1)	2.618 (1)	154 (2)
O7—H7···O6	0.85 (1)	1.79 (1)	2.595 (1)	156 (2)
C7—H7A···O8^i^	0.95	2.48	3.4222 (12)	173
C9—H9···O2^ii^	0.95	2.35	3.2614 (12)	160
C12—H12B···O2^iii^	0.98	2.37	3.3326 (13)	166
C18—H18···O4^iv^	0.95	2.47	3.3904 (12)	163
C20—H20···O6^iii^	0.95	2.27	3.1995 (12)	166

Symmetry codes: (i) *x*, −*y*+1/2, *z*−1/2; (ii) −*x*+1, *y*+1/2, −*z*+3/2; (iii) −*x*+2, *y*+1/2, −*z*+3/2; (iv) *x*, −*y*+1/2, *z*+1/2.
